# Genome-Wide Association Studies for Growth Curves in Meat Rabbits Through the Single-Step Nonlinear Mixed Model

**DOI:** 10.3389/fgene.2021.750939

**Published:** 2021-10-08

**Authors:** Yonglan Liao, Zhicheng Wang, Leonardo S. Glória, Kai Zhang, Cuixia Zhang, Rui Yang, Xinmao Luo, Xianbo Jia, Song-Jia Lai, Shi-Yi Chen

**Affiliations:** ^1^ Farm Animal Genetic Resources Exploration and Innovation Key Laboratory of Sichuan Province, Sichuan Agricultural University, Chengdu, China; ^2^ Laboratory of Animal Science, State University of Northern of Rio de Janeiro, Campos dos Goytacazes, Brazil; ^3^ Sichuan Academy of Grassland Sciences, Chengdu, China; ^4^ Animal Breeding and Genetics Key Laboratory of Sichuan Province, Sichuan Animal Science Academy, Chengdu, China

**Keywords:** GWAS, longitudinal data, genomic analysis, NMM, body weight

## Abstract

Growth is a complex trait with moderate to high heritability in livestock and must be described by the longitudinal data measured over multiple time points. Therefore, the used phenotype in genome-wide association studies (GWAS) of growth traits could be either the measures at the preselected time point or the fitted parameters of whole growth trajectory. A promising alternative approach was recently proposed that combined the fitting of growth curves and estimation of single-nucleotide polymorphism (SNP) effects into single-step nonlinear mixed model (NMM). In this study, we collected the body weights at 35, 42, 49, 56, 63, 70, and 84 days of age for 401 animals in a crossbred population of meat rabbits and compared five fitting models of growth curves (Logistic, Gompertz, Brody, Von Bertalanffy, and Richards). The logistic model was preferably selected and subjected to GWAS using the approach of single-step NMM, which was based on 87,704 genome-wide SNPs. A total of 45 significant SNPs distributed on five chromosomes were found to simultaneously affect the two growth parameters of mature weight (A) and maturity rate (K). However, no SNP was found to be independently associated with either A or K. Seven positional genes, including *KCNIP4*, *GBA3*, *PPARGC1A*, *LDB2*, *SHISA3*, *GNA13*, and *FGF10*, were suggested to be candidates affecting growth performances in meat rabbits. To the best of our knowledge, this is the first report of GWAS based on single-step NMM for longitudinal traits in rabbits, which also revealed the genetic architecture of growth traits that are helpful in implementing genome selection.

## Introduction

The domestic rabbit (*Oryctolagus cuniculus*) is an important livestock species in China and has been intensively raised for producing meat, wool, and fur. The most commonly raised type is meat rabbits in China, and the rabbit meat production reached 849,150 tons in 2016, which almost accounted for about 60% of global production (Li et al., 2018). However, progresses on genetic selection and improvement in rabbits have obviously lagged behind in comparison with other livestock species; therefore, the Chinese meat rabbit industry is still largely depending on these imported breeds, such as the Hyla, Hycole, and Hyplus rabbits from France (Qin, 2019). One of the important reasons is the serious lack of relevant studies conducted in rabbits, such as the genome-wide association studies (GWAS) and genomic selection (GS) for the economically important traits. Recently, some pioneer studies were published about the GWAS (Sosa-Madrid et al., 2020; Yang et al., 2020; Bovo et al., 2021) and GS (Chen et al., 2021; Helal et al., 2021; Mancin et al., 2021) in rabbits.

Growth is a complex and economically important trait with moderate to high estimates of heritability in rabbits (Akanno and Ibe, 2005; Dige et al., 2012; Soliman et al., 2014). In contrast to traits that are collected at a single time point (such as litter size and carcass performances), growth must be described by the longitudinal data repeatedly measured over multiple time points. Therefore, the relevant genetic studies on growth in livestock can be implemented through different approaches. The first approach is to select one or a few representative time points and subject them to separate analysis; for instance, the GWAS of growth traits were separately performed among multiple time points of age in meat rabbits (Yang et al., 2020). The second approach is to fit growth curves using nonlinear regression models and obtain the growth curve parameters (such as mature weight and maturity rate), and subsequently, these derived parameters are used as the pseudo-phenotypes for association analysis. This is the classical two-step method and has been commonly found in literature, such as the studies in beef cattle (Crispim et al., 2015; Duan et al., 2021). Furthermore, the two-step method could be followed by an additional step of multi-trait meta-analysis to indirectly combine the multiple parameters together (Duan et al., 2021). Recently, Silva et al. (2017) proposed an alternative modeling framework to integrate the fitting of growth curves and estimation of single-nucleotide polymorphism (SNP) effects simultaneously under nonlinear mixed model (NMM), which was applied to pigs and revealed to have the advantages of higher statistical power and joint modeling of residual effects in comparison with the two-step method. To our best knowledge, this single-step method has not yet been applied to GWAS of growth curves in rabbits.

In this context, we collected the individual growth records from weaning at 35 days of age (DOA) to finishing at 84 DOA in a commercial crossbred population of meat rabbits. Subsequently, the fitting of growth curves and GWAS were simultaneously analyzed using a single-step NMM to identify the prospective candidate variants, genes, and biological processes associated with growth trajectory. These results could be helpful in understanding the biological mechanisms underlying growth and implementing GS of growth traits in rabbits.

## Materials and Methods

### Animals and Phenotypes

One commercial crossbred population of meat rabbits, by crossing 22 Kangda5 rabbits (♂) with 53 Californian rabbits (♀), was subjected to collection of phenotypic records, which was described in our previous study (Yang et al., 2020). In brief, individual body weight (BW) was initially measured for 461 rabbits at seven time points, including 35, 42, 49, 56, 63, 70, and 84 DOA, respectively. At each time point, the phenotypic records were set to missing values if they deviated by more than three standard deviations (SD) from the population mean. As the short time intervals were measured, the individual BW was allowed to be slightly decreased (<5%) between two consecutive time points; otherwise, the latter record was set to missing value. The individuals that have more than two missing values at the seven time points were also removed, after which 405 individuals remained. No pedigree information is available for this population.

### Genotypes and Quality Controls

For the initial SNP set that was generated from specific-locus amplified fragment sequencing approach (Yang et al., 2020), we reapplied more strict criterion of quality control (QC) using the filtering expression of “QualByDepth (QD) < 2.0 || FisherStrand (FS) > 60.0 || RMSMappingQuality (MQ) < 40.0” intrinsically implemented in GATK software v4.2 (McKenna et al., 2010). A total of 6,721,762 SNPs were obtained and subjected to additional QC steps using PLINK software v1.9 (Chang et al., 2015), which required the genotype missing rate lower than 0.1, individual missing rate lower than 0.2, minor allele frequency (MAF) higher than 0.05, and no extreme deviation from Hardy–Weinberg equilibrium (i.e., only retained SNPs with *p* > 1.0E−08). Furthermore, the missing genotypes were imputed using Beagle software v5.1 with default parameters (Browning et al., 2018). Using PLINK software v1.9 (Chang et al., 2015), the tightly linked SNPs were further discarded if the linkage disequilibrium (LD) values were higher than 0.9. Finally, 87,704 SNPs were used for GWAS among 401 individuals (215 males and 186 females), and these SNPs were distributed among all 21 rabbit autosomes (OCU). To investigate population structure, principal component analysis (PCA) was performed based on the finally included genotypes using PLINK software v1.9 (Chang et al., 2015).

### Modeling of Growth Curves

Five nonlinear regression models were evaluated for fitting the growth curves (Koya and Goshu, 2013), including the logistic of $w_{t}=A{\left[1+b\exp\left(-Kt\right)\right]}^{-1}$ , Gompertz of $w_{t}=A\exp\left[-b\exp\left(-Kt\right)\right]$ , Brody of $w_{t}=A\left[1-b\exp\left(-Kt\right)\right]$ , Von Bertalanffy of $w_{t}=A{\left[1-b\exp\left(-Kt\right)\right]}^{3}$ , and Richards of $w_{t}=A{\left[1\pm b\exp\left(-Kt\right)\right]}^{m}$ . Among them, $w_{t}$ is the individual BW at time t ; and the parameter A , K , and b are the mature weight, maturity rate, and time-scale parameter, respectively. Furthermore, m is the shape parameter in Richards model. The fitting of growth curves was performed using the *nlme* package of R (Heisterkamp et al., 2017), and the model with the best goodness of fit was selected according to the Akaike information criterion (AIC) (Akaike, 1974) and Bayesian information criterion (BIC) (Schwarz, 1978).

### Genome-Wide Association Studies

The logistic was selected as best model (see *Results* section) and therefore used for the GWAS of growth curves through single-step NMM following Silva et al. (2017). This method fitted the two biological meaningful parameters of growth curves (i.e., A and K ) through the NMM. Therefore, the null model (M0) without considering SNP effect was defined as follows:

$$w_{it}=\frac{\mu_{A}+Sex+PC+\varepsilon_{A_{i}}}{1+\mu_{b}\exp\left[-\left(\mu_{K}+Sex+PC+\varepsilon_{K_{i}}\right)t\right]}+e_{it},$$

where $w_{it}$ is the BW of the individual i at time t ; $\mu_{A}$ , $\mu_{K}$ , and $\mu_{b}$ are the general means for parameter A , K , and b , respectively; Sex and PC are the fixed effects of sex and five principal components (PC) of genotype matrix ( $PC_{1}$ , $PC_{2}$ , $PC_{3}$ , $PC_{4}$ , and $PC_{5}$ ), respectively; $\varepsilon_{A_{i}}$ and $\varepsilon_{K_{i}}$ are the specific residuals for parameter A and K of individual i ; and $e_{it}$ is a general residual of individual i at time t , assumed with $e_{it}∼N\left(0,\sigma_{e}^{2}\right)$ . The assumed (co)variance structures of $\varepsilon_{A_{i}}$ and $\varepsilon_{K_{i}}$ were as follows:

$$\left[\begin{array}{c} \varepsilon_{A_{i}}\\ \varepsilon_{K_{i}} \end{array}\right]∼N\left(0,\left[\begin{array}{cc} \sigma_{A}^{2} & \sigma_{A,K}\\ \sigma_{A,K} & \sigma_{K}^{2} \end{array}\right]\right),$$

where $\sigma_{A}^{2}$ , $\sigma_{K}^{2}$ , and $\sigma_{A,K}$ are the specific residual variances and covariance for parameter A and K .

According to Silva et al. (2017), the SNP effects could be further integrated into the null model through three different ways. First, the SNP effects are assumed to simultaneously affect both A and K parameters, and this full model (M1) was given as follows:

$$w_{it}=\frac{\mu_{A}+Sex+PC+SNP+\varepsilon_{A_{i}}}{1+\mu_{b}\exp\left[-\left(\mu_{K}+Sex+PC+SNP+\varepsilon_{K_{i}}\right)t\right]}+e_{it},$$

where SNP is fixed effects. Alternatively, the SNP effects independently affect either A (M2) or K (M3), and their models were, respectively, given as follows:

$$w_{it}=\frac{\mu_{A}+Sex+PC+SNP+\varepsilon_{A_{i}}}{1+\mu_{b}\exp\left[-\left(\mu_{K}+Sex+PC+\varepsilon_{K_{i}}\right)t\right]}+e_{it},$$

$$w_{it}=\frac{\mu_{A}+Sex+PC+\varepsilon_{A_{i}}}{1+\mu_{b}\exp\left[-\left(\mu_{K}+Sex+PC+SNP+\varepsilon_{K_{i}}\right)t\right]}+e_{it}.$$

The fitting of these four NMM was performed using the *nlme* package of R (Heisterkamp et al., 2017). Based on the likelihood ratio test (LRT), the statistical significance of SNP effects could be deduced by comparing the specific alternative hypotheses (i.e., the model of M1, M2, or M3) with the null hypothesis of M0, respectively. The derived LRT statistics are assumed to follow $\chi^{2}$ distribution with n degrees of freedom, where n is the difference of the number of parameters between the two models compared. To address the multiple comparison problem, the false discovery rate (FDR) method was employed for computing the adjusted *p*-values using the *qvalue* package of R (Storey et al., 2004). As a result, SNP was statistically significant with FDR <0.05.

### Functional Analysis

In this study, the QTLs were empirically defined as chromosomal regions of ±100 kb around the significant SNPs (i.e., a total of 200-kb genomic region was selected). The candidate genes within QTL, including protein encoding and long non-coding RNAs (lncRNA), were retrieved using the biomaRt R package (Smedley et al., 2015). The OryCun2.0 assembly was used as the reference genome (https://www.ncbi.nlm.nih.gov/genome/?term=rabbit). For all the candidate genes, the functional enrichments were conducted using the DAVID tool (Huang et al., 2009), including the Gene Ontology (GO) terms (The Gene Ontology Consortium, 2019) and Kyoto Encyclopedia of Genes and Genomes (KEGG) pathway (Kanehisa et al., 2019). The default parameters and method of multiple testing correction were used for computing *p*–values, and the threshold of 0.05 was set.

## Results

### Descriptive Statistics

For the 401 finally included individuals, the descriptive statistics of BW at the seven time points are shown in Table 1, and their normal distributions were visually checked at every time points (Supplementary Figure S1). The 87,704 SNPs were distributed among 21 autosomes with the mean (±SD) of 24,099 ± 59,103 bp for their pairwise physical distances and 0.256 ± 0.133 for MAF, respectively (Supplementary Figure S2).

**TABLE 1:** The descriptive statistics of body weight at the seven time points.

Days of age	Number of records	Body weight (g)			
		Min	Max	Mean	SD
35	399	456	1,120	788.05	122.65
42	398	741	1,327	1,012.73	112.42
49	401	874	1,657	1,244.87	136.78
56	401	972	2,005	1,474.96	179.96
63	381	974	2,354	1,706.96	235.45
70	371	1,050	2,726	1,948.76	285.08
84	363	1,487	2,888	2,238.13	285.29

Note. SD, standard deviation.

### Fitted Growth Curves

**FIGURE 1:**
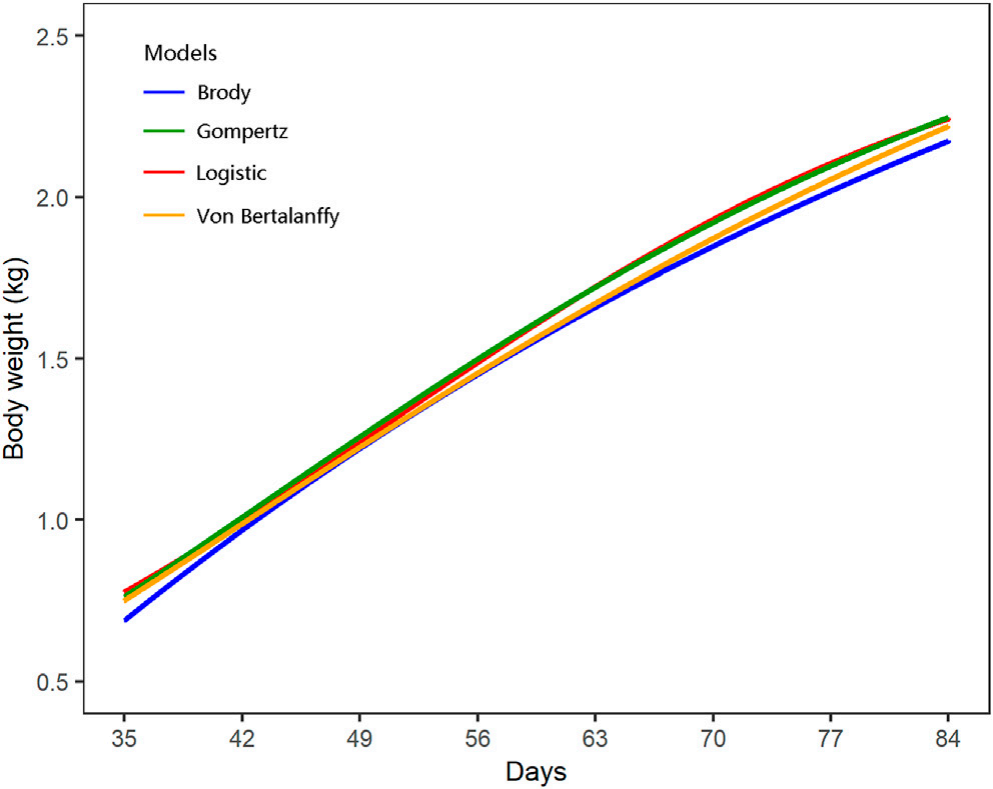
Fitting of growth curves using the four nonlinear regression models.

The growth curves of all individuals were successfully fitted using the four candidate models of logistic (AIC = 35,667.62 and BIC = 35,697.32), Gompertz (AIC = 35,699.36 and BIC = 35,729.06), Brody (AIC = 35,830.33 and BIC = 35,860.03), and Von Bertalanffy (AIC = 37,737.74 and BIC = 37,767.44), whereas the model of Richards did not converge and was therefore excluded for comparison (Figure 1; Supplementary Table S1). Among the four NMM successfully fitted, the logistic model showed the best goodness of fit with the lowest values of AIC and BIC, and was selected for the following GWAS. The estimates of parameter A and K of the logistic growth curves were 2,615.45 and 0.054, respectively. Furthermore, the growth curves of females and males were separately fitted, which also supported the logistic model having the best goodness of fit and similar growth parameters (Supplementary Table S1). There were only small differences for the A and K parameters estimated between males and females.

### Association Analyses

**FIGURE 2:**
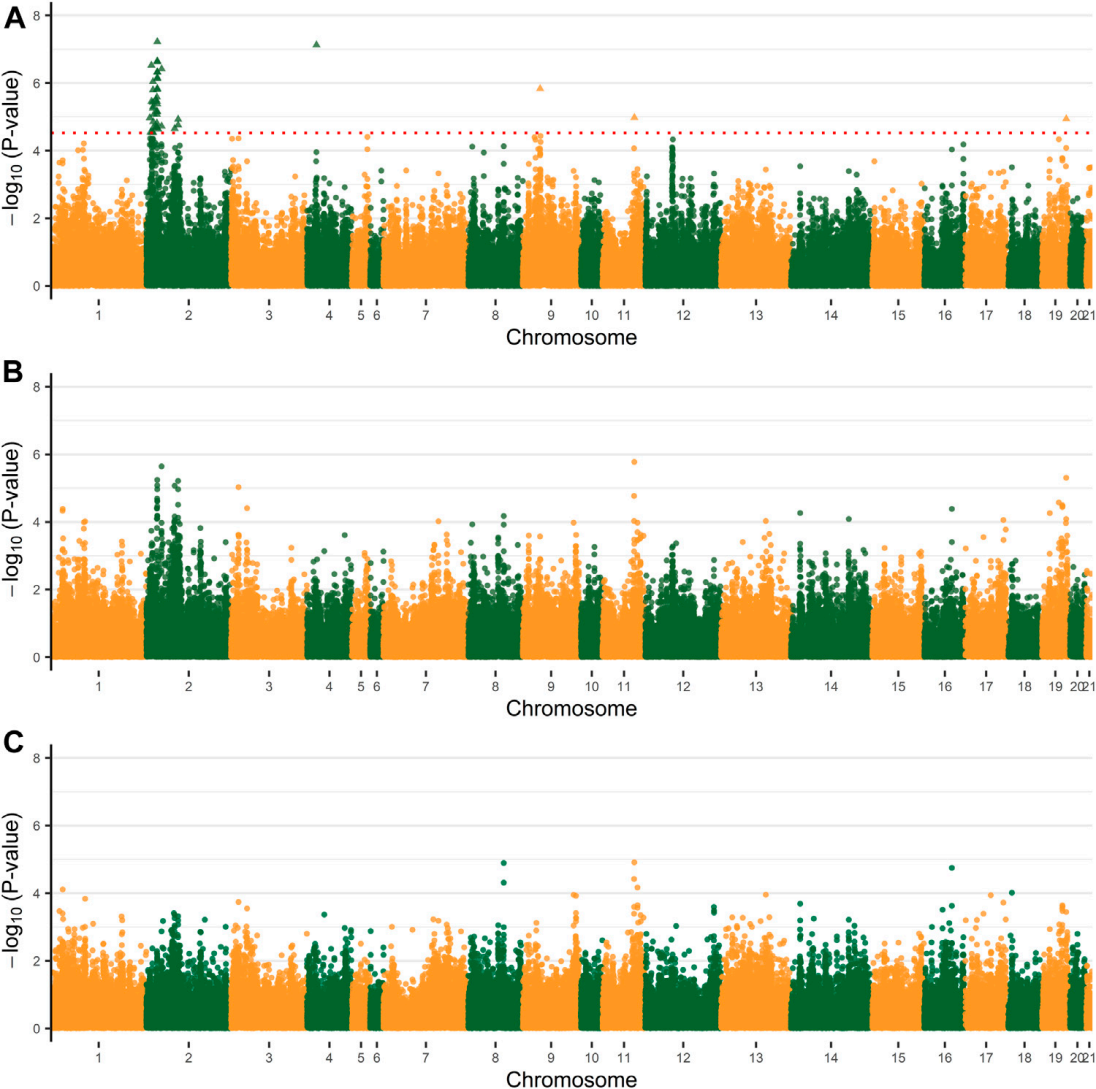
Manhattan plots of genome-wide association analysis (GWAS) for mature weight (A), maturity rate (K). **(A)** The Manhattan plot of both the parameter A and K. **(B)** The Manhattan plot of the parameter A. **(C)** The Manhattan plot of the parameter K. The dashed line of red indicates a 5% FDR-corrected threshold and the significant single-nucleotide polymorphisms (SNPs) are represented by triangles.

Based on the PCA results (Supplementary Figure S3), no obvious population stratification was observed in this population studied. The first five PCs explained about 58.9% of total variability, which were included in the NMM as fixed effects with alleviated convergence problems. A total of 45 significant SNPs were revealed to simultaneously affect both parameter A and K, which were distributed among five chromosomes, OCU2, OCU4, OCU9, OCU11, and OCU19 (Figure 2; Table 2). Among them, the highest numbers of significant SNPs were observed on OCU2 (N = 41), and the three most significant SNPs were located on OCU2 (*p* = 5.98E−08), OCU4 (*p* = 7.51E−08), and OCU2 (*p* = 2.22E−07), respectively. All the 45 significant SNPs were clustered into 24 QTLs, and three of their QTLs (OCU2: 13.67–13.99 Mb, OCU2: 21.86–22.25 Mb, OCU2: 22.45–22.77 Mb) were identified based on three or more SNPs (Table 2). When considering the SNP effect separately for either parameter A or K, no significant SNP was identified at the predefined threshold (Figure 2). However, some suggestive associations were also observed (with *p* lower or close to 1.0E−05), such as the SNPs on OCU2, OCU3, OCU11, and OCU19 for parameter A, and OCU8 and OCU11 for parameter K.

**TABLE 2:** Significant SNPs, QTLs, and candidate genes simultaneously affect both parameter A and K of the logistic growth curve in rabbits.

Chromosomes	SNP position (bp)	*p*	Locations	QTL region (bp)	Candidate genes
OCU2	7,392,553	1.06E−05	Intron	7,292,553–7,492,553	*LDB2*
	8,791,893	2.88E−05	Intergenic	8,691,893–8,891,893	ENSOCUG00000035404^a^
	10,501,993	3.52E−06	Intergenic	10,401,993–10,691,957	None
	10,591,957	2.98E−07	Intergenic		
	11,378,860	2.94E−05	Intron	11,278,860–11,478,860	*PACRGL* and *KCNIP4*
	13,386,997	5.63E−06	Intergenic	13,286,997–13,486,997	*GBA3*
	13,488,329	3.69E−06	Intergenic	13,388,329–13,588,329	ENSOCUG00000031640^a^
	13,770,022	2.93E−05	Intergenic	13,670,022–13,985,731	ENSOCUG00000031081^a^ and ENSOCUG00000034770^a^
	13,775,984	9.13E−07	Intergenic		
	13,847,789	1.60E−06	Intergenic		
	13,885,731	2.86E−05	Intergenic		
	14,218,502	2.09E−05	Intergenic	14,118,502–14,318,502	None
	14,381,825	1.39E−05	Intergenic	14,281,825–14,488,318	*PPARGC1A*
	14,388,318	4.97E−06	Intergenic		
	15,540,704	8.39E−06	Intergenic	15,440,704–15,640,704	*CCDC149*, *LGI2*, ENSOCUG00000029972^a^, ENSOCUG00000037097^a^, and ENSOCUG00000037279^a^
	19,348,590	2.04E−05	Intergenic	19,248,590–19,448,590	None
	19,519,932	3.07E−06	Intergenic	19,419,932–19,619,932	None
	21,766,582	1.53E−05	Intergenic	21,666,582–21,866,582	None
	21,961,157	2.03E−05	Intergenic	21,861,157–22,251,345	ENSOCUG00000039621^a^
	21,961,341	2.61E−06	Intergenic		
	21,991,030	8.43E−06	Intergenic		
	22,076,402	7.17E−06	Intergenic		
	22,080,992	2.06E−05	Intergenic		
	22,082,402	6.76E−06	Intergenic		
	22,151,345	3.50E−06	Intergenic		
	22,287,659	1.54E−06	Intergenic	22,187,659–22,387,659	None
	22,552,417	5.98E−08	Intergenic	22,452,417–22,774,072	ENSOCUG00000032798^a^
	22,559,081	4.80E−07	Intergenic		
	22,559,709	2.22E−07	Intergenic		
	22,559,791	4.18E−06	Intergenic		
	22,585,579	2.37E−07	Intergenic		
	22,597,775	7.40E−07	Intergenic		
	22,628,803	6.92E−07	Intergenic		
	22,632,153	1.45E−06	Intergenic		
	22,674,072	4.67E−07	Intergenic		
	24,405,104	2.30E−05	Intergenic	24,305,104–24,505,104	None
	31,443,212	1.89E−05	Intergenic	31,343,212–31,566,952	*ATP8A1* and *SHISA3*
	31,466,952	3.82E−07	3′-UTR		
	58,261,249	2.21E−05	Intergenic	58,161,249–58,361,249	None
	65,306,234	1.18E−05	Intergenic	65,206,234–65,496,063	*LOC100358067*
	65,396,063	1.74E−05	Intergenic		
OCU4	19,121,968	7.51E−08	Intergenic	19,021,968–19,221,968	ENSOCUG00000038375^a^
OCU9	35,523,218	1.48E−06	Intron	35,423,218–35,623,218	*TAFA1*
OCU11	64,951,640	1.05E−05	Intergenic	64,851,640–65,051,640	ENSOCUG00000037935^a^, ENSOCUG00000029125, ENSOCUG00000037904^a^, and *FGF10*
OCU19	52,159,278	1.15E−05	Intron	52,059,278–52,259,278	*RGS9*, ENSOCUG00000036189, *GNA13*, *AMZ2*, *SLC16A6*, and *ARSG*

a lncRNA; 3′'-UTR, 3′-untranslated region; SNP, single-nucleotide polymorphism.

### Candidate Genes and Functional Analyses

Within the 24 candidate QTLs regarding the significant SNP effects on both parameter A and K, a total of 19 protein-coding and 12 lncRNA positional candidate genes were identified (Table 2). Of these, four [LIM domain-binding 2 (*LDB2*), potassium voltage-gated channel interacting protein 4 (*KCNIP4*), TAFA chemokine like family member 1 (*TAFA1*), and G protein subunit alpha 13 (*GNA13*)] and one [ATPase phospholipid transporting 8A1 (*ATP8A1*)] candidate genes were found to have the significant SNPs located on intron and 3′-untranslated region (3′-UTR), respectively. Furthermore, three protein-coding genes located on OCU2 were supported by more than one significant SNPs, including the peroxisome proliferator-activated receptor gamma coactivator-1 alpha (*PPARGC1A*), ATPase phospholipid transporting 8A1 (*ATP8A1*), and shisa family member 3 (*SHISA3*) gene. Two lncRNA genes (ENSOCUG00000039621 and ENSOCUG00000032798) had seven and nine significant SNPs that were located on intergenic regions, respectively. The detailed information of these positional candidate genes is shown in Supplementary Table S2.

For these positional candidate genes, 19 biological processes of GO terms were significantly enriched (*p* < 0.05, Supplementary Table S3). However, no significant KEGG pathway was found. Four genes of *GNA13*, *ATP8A1*, *LDB2*, and fibroblast growth factor 10 (*FGF10*) were observed in eight GO terms that were mainly involved in the cell development, such as the biological processes of “regulation of cell migration” and “regulation of cell motility.” Furthermore, both *LDB2* and *FGF10* were enriched in the five GO terms that have the functional implications into growth, such as the biological processes of “somatic stem cell population maintenance” and “maintenance of cell number.”

## Discussion

Growth traits have considerable economic implications in meat rabbit industry. For Gabali rabbits in Egypt, the heritability estimates were 0.19, 0.23, 0.16, and 0.14 for BW at 4, 8, 12, and 16 weeks of age (Soliman et al., 2014), which suggested a moderate heritability for these growth traits. The estimated heritability of individual BW ranged from 0.11 at 9 weeks of age to 0.43 at 6 weeks of age in New Zealand White and Dutch breeds of rabbits (Akanno and Ibe, 2005). The moderate to high heritability (from 0.266 to 0.540) was similarly estimated using both Sire Model and Animal Model in New Zealand White rabbits (Dige et al., 2012). Abou Khadiga et al. (2008) conducted the genetic evaluation in crossbred population of Spanish synthetic maternal line V and Egyptian Baladi Black, and found that growth traits were significantly affected by direct genetic effects. Furthermore, the genotype × environment interaction was also observed for affecting growth performances in growing rabbits (Zeferino et al., 2011). Together, these studies indicated that the improvement of growth traits by genetic selection is much feasible in rabbits. However, the relevant studies in rabbits, such as genomic evaluation and GWAS, have largely lagged behind in comparison with other livestock species (Jonas and Koning, 2015). Therefore, in this study, we performed the association analyses for individual BW at different growth time points using the genome-wide variants. As a relatively limited number of rabbits were included in the present study, however, the increased detection power of GWAS would be expected using larger datasets in future studies.

Like milk production traits in dairy livestock, the individual growth has been preferably described by longitudinal records measured over multiple time points. In practices, the phenotypic records at one or a few time points could be representatively selected and analyzed. However, an alternative approach is to fit the whole growth trajectory using nonlinear regression models and then use the derived model parameters for describing individual growth performance. In an early study (Ptak et al., 1994), three nonlinear models of Von Bertalanffy, Gompertz, and logistic were compared for fitting the growth in purebred and crossbred rabbits, and found that the Von Bertalanffy gave the best fit. The Gompertz growth curves were fitted and used in analyzing the effect of selection for growth rate on growth curves in rabbits (Blasco et al., 2003). Recently, Ding et al. (2019) fitted the growth curves using the logistic, Gompertz, and Von Bertalanffy models for crossbred population of California rabbit × New Zealand white rabbit and suggested that the most accurate model was logistic. In this study, the logistic was chosen as the best model to describe the growth trajectory of our crossbred population that was generated by crossing Kangda5 rabbits with Californian rabbits, which was consistent with the results of Ding et al. (2019). Therefore, the selection of the best model to fit the growth curve in rabbits would be breed or population dependent, which should be specifically compared in each study.

In livestock and poultry, mature weight and maturity rate are the two important parameters for describing growth performance; some individuals have higher maturity rate but smaller mature weight, and vice versa. Therefore, to identify genes or causal mutations independently affecting the mature weight and maturity rate is essential for implementing precision improvement of genetic selection. Using the estimated growth parameters as pseudo-phenotypes in GWAS of growth traits in Brahman cattle, a large number of significant SNPs were identified to be associated with mature weight and maturity rate, respectively (Crispim et al., 2015). A similar GWAS was recently reported for growth traits of Chinese Simmental beef cattle, which also revealed different SNPs for the two parameters (Duan et al., 2021). Silva et al. (2017) proposed an alternative method to combine the fitting of growth curves and estimation of SNP effects into one single-step NMM, and to apply to growth traits in pigs with an improved statistical power observed. In this study, we also employed the single-step NMM approach for GWAS of growth traits in a crossbred population of rabbits and found that all the significant SNPs simultaneously affected the two parameters of mature weight and maturity rate. The absence of SNPs independently associated with either mature weight or maturity rate would indicate the specific genetic architecture of growth performance for this studied population. Also, the number of significant SNPs identified in this study was also higher than our former observation (Yang et al., 2020) that was alternatively performed through the separate association analysis with BW at different time points. However, no growth curve parameter was estimated and used as pseudo-phenotype of association analysis by Yang et al. (2020), which would disable the direct comparison.

Around the significant SNPs identified in this study, we found some candidate genes that have the functional implications on growth traits in literature. Among them, the *KCNIP4*, a member of the family of voltage-gated potassium channel-interacting proteins, was found to be located within the QTL between the 21 and 67 cM regions of chromosome 6 that was associated with birth weight in Zandi sheep (Esmailizadeh, 2010; Mohammadi et al., 2020). Pasandideh et al. (2018) also suggested that the *KCNIP4* gene was involved in the regulation of muscle growth and fat deposition in sheep. In chicken, Jin et al. (2015) found that *KCNIP4* was located nearest to a significant SNP associated with the BW of 10 and 14 weeks of age. A nearby gene of *GBA3* (glucosylceramidase beta 3) is related to hydrolyze beta-galactose and beta-glucose (Dekker et al., 2011), and was further found to be significantly associated with BW traits in sheep (Al-Mamun et al., 2015). In this study, one significant SNP was detected in the intron of *LDB2*, which is a transcriptional regulator (Johnsen et al., 2009) and was found to affect the growth traits of BW and average daily gain in chicken (Gu et al., 2011; Wang et al., 2019) and of BW in Nanjiang Yellow sheep (Guo et al., 2018).

Another important candidate gene is *PPARGC1A*, which was found in pigs to regulate the lipid deposition (Li et al., 2014b), composition of muscle fiber (Lee et al., 2012), and abdominal fat content (Stachowiak et al., 2007). Several SNPs have been identified in *PPARGC1A* gene to be associated with adult BW and average daily gain in Nanyang cattle (Li et al., 2014a), yearling weight in Nelore cattle (Fonseca et al., 2015), and birth weight and calf birth weight in Iranian Holstein cattle (Pasandideh, 2020). Furthermore, Chen et al. (2020) found a 17-bp InDel mutation within the 11th intron of *PPARGC1A* gene in sheep, which was associated with the BW. Both *GNA13* and *SHISA3* could affect individual growth as they are involved in the biological regulation of osteoclastogenesis and bone development (Wu et al., 2017; Murakami et al., 2019). Furthermore, we observed that the *FGF10* gene located on OCU11 was significantly associated with both mature weight and maturity rate. Previous studies revealed that *FGF10* could promote the proliferation and differentiation of adipocyte through the Ras/MAKP pathway (Konishi et al., 2006), and regulate adipogenesis in muscle tissue of goats (Xu et al., 2018) and Tibetan chickens (Zhang et al., 2018). We did not find the relevant publication in literature about functional implications for the 12 candidate lncRNA genes found in this study.

The post-GWAS functional studies are necessary for fine mapping the causal genetic variants and dissecting the underlying biological mechanism (Gallagher and Chen-Plotkin, 2018). Therefore, these candidate genes found in this study could be preferably selected in future studies to investigate their functional mechanisms affecting the individual growth in rabbits. On the other hand, these significant SNPs and genomic regions could be incorporated into the genomic prediction models with an improved accuracy, by using the weighted genomic best linear unbiased prediction (Zhang et al., 2016) or Bayesian (van den Berg et al., 2020) approaches.

## Conclusion

In the crossbred population of meat rabbits, we employed the nonlinear mixed model to simultaneously fit growth curves and estimate SNP effects at the genome-wide level. The significant SNPs on five chromosomes (OCU2, OCU4, OCU9, OCU11, and OCU19) were found to simultaneously affect the mature weight and maturity rate, which further revealed some suggestive candidate genes, including the *KCNIP4*, *GBA3*, *PPARGC1A*, *LDB2*, *SHISA3*, *GNA13*, and *FGF10*. These obtained results are useful to increase our knowledge about growth mechanisms in rabbits, and could be used for improving the accuracy of genomic selection in this population.

## Data Availability

The original contributions presented in the study are included in the article/Supplementary Material, further inquiries can be directed to the corresponding authors.
