# Supplementary material for: Genome-Wide Association Studies for Growth Curves in Meat Rabbits Through the Single-Step Nonlinear Mixed Model
Source: Front Genet. 2021 Oct 8;12:750939. doi: 10.3389/fgene.2021.750939 (PMC8531506; doi:10.3389/fgene.2021.750939)
Supplement: Supplementary file 1 [file DataSheet1.docx]

**Supplementary Table S1. Estimated values and goodness of fit in four growth curve models.**

| **Model** | **Males and females** | | | | **Males** | | | | **Females** | | | |
| --- | --- | --- | --- | --- | --- | --- | --- | --- | --- | --- | --- | --- |
|  | **A** | **K** | **AIC** | **BIC** | **A** | **K** | **AIC** | **BIC** | **A** | **K** | **AIC** | **BIC** |
| **Logistic** | 2,615.45 | 0.054 | 35,667.62 | 35,697.32 | 2,618.13 | 0.054 | 19,200.47 | 19,227.05 | 2,613.18 | 0.055 | 16,464.71 | 16,490.57 |
| **Gompertz** | 2,925.96 | 0.033 | 35,699.36 | 35,729.06 | 2,922.28 | 0.033 | 19,216.75 | 19,243.33 | 2,932.02 | 0.033 | 16,480.24 | 16,506.10 |
| **Brody** | 3,679.42 | 0.014 | 35,830.33 | 35,860.03 | 3,641.39 | 0.015 | 19,283.65 | 19,310.23 | 3,736.87 | 0.014 | 16,544.32 | 16,570.18 |
| **Von Bertalanffy** | 3,333.98 | 0.023 | 37,737.74 | 37,767.44 | 3,355.24 | 0.023 | 20,205.57 | 20,232.15 | 3,309.84 | 0.024 | 17,537.30 | 17,563.16 |
| **Richards** | The model was a singular fitting state and did not converge successfully. | | | | | | | | | | | |

**Supplementary Table S2. The detailed description of the protein-coding gene for the significant SNP simultaneously affects both A and K parameters.**

| **Ensembl ID** | **Location (bp)** | **Candidate genes** | |
| --- | --- | --- | --- |
|  |  | **Symbol** | **Full names** |
| ENSOCUG00000001594 | OCU19: 52,215,848-52,227,671 | *SLC16A6* | Solute carrier family 16 member 6 |
| ENSOCUG00000002186 | OCU2: 11,313,619-11,333,944 | *PACRGL* | PARK2 coregulated like |
| ENSOCUG00000002189 | OCU2: 11,334,956-12,545,766 | *KCNIP4* | Potassium voltage-gated channel interacting protein 4 |
| ENSOCUG00000003079 | OCU19: 51,999,202-52,088,717 | *RGS9* | Regulator of G-protein signaling 9 |
| ENSOCUG00000004764 | OCU2: 15,572,880-15,598,150 | *LGI2* | Leucine rich repeat LGI family member 2 |
| ENSOCUG00000005568 | OCU2: 13,239,653-13,362,378 | *GBA3* | Glucosylceramidase beta 3 |
| ENSOCUG00000007463 | OCU2: 15,397,891-15,499,403 | *CCDC149* | Coiled-coil domain containing 149 |
| ENSOCUG00000007640 | OCU19: 52,119,221-52,160,069 | *GNA13* | G protein subunit alpha 13 |
| ENSOCUG00000008499 | OCU11: 64,991,608-65,079,991 | *FGF10* | Fibroblast growth factor 10 |
| ENSOCUG00000011108 | OCU2: 31,462,723-31,466,010 | *SHISA3* | Shisa family member 3 |
| ENSOCUG00000013152 | OCU2: 31,451,308-31,734,499 | *ATP8A1* | ATPase phospholipid transporting 8A1 |
| ENSOCUG00000014668 | OCU2: 14,349,580-14,501,645 | *PPARGC1A* | PPARG coactivator 1 alpha |
| ENSOCUG00000014789 | OCU19: 52,193,781-52,203,777 | *AMZ2* | Archaelysin family metallopeptidase 2 |
| ENSOCUG00000015499 | OCU2: 7,063,584-7,464,403 | *LDB2* | LIM domain binding 2 |
| ENSOCUG00000017402 | OCU19: 52,250,502-52,337,251 | *ARSG* | Arylsulfatase G |
| ENSOCUG00000026451 | OCU2: 65,288,315-65,290,567 | *LOC100358067* | Cytosolic beta-glucosidase |
| ENSOCUG00000031560 | OCU9: 35,298,296-35,863,079 | *TAFA1* | TAFA chemokine like family member 1 |
| ENSOCUG00000029125 | OCU11: 64,871,108-64,872,133 | - | ENSOCUG00000029125 |
| ENSOCUG00000036189 | OCU19: 52,117,642-52,119,972 | - | ENSOCUG00000036189 |

**Supplementary Table S3. The functions of candidate genes for the SNP simultaneously affecting both A and K parameters.**

| **Category** | **Term ID** | **Term name** | **Gene list** | ***P*** |
| --- | --- | --- | --- | --- |
| GOTERM_BP_FAT | GO:0030334 | Regulation of cell migration | *GNA13*, *ATP8A1*, *LDB2*, *FGF10* | 3.83E-03 |
| GOTERM_BP_FAT | GO:2000145 | Regulation of cell motility | *GNA13*, *ATP8A1*, *LDB2*, *FGF10* | 4.22E-03 |
| GOTERM_BP_FAT | GO:0040012 | Regulation of locomotion | *GNA13*, *ATP8A1*, *LDB2*, *FGF10* | 5.00E-03 |
| GOTERM_BP_FAT | GO:0051270 | Regulation of cellular component movement | *GNA13*, *ATP8A1*, *LDB2*, *FGF10* | 5.54E-03 |
| GOTERM_BP_FAT | GO:0051674 | Localization of cell | *GNA13*, *ATP8A1*, *LDB2*, *FGF10* | 2.41E-02 |
| GOTERM_BP_FAT | GO:0048870 | Cell motility | *GNA13*, *ATP8A1*, *LDB2*, *FGF10* | 2.41E-02 |
| GOTERM_BP_FAT | GO:0035019 | Somatic stem cell population maintenance | *LDB2*, *FGF10* | 2.59E-02 |
| GOTERM_BP_FAT | GO:0040011 | Locomotion | *GNA13*, *ATP8A1*, *LDB2*, *FGF10* | 3.32E-02 |
| GOTERM_BP_FAT | GO:0006928 | Movement of cell or subcellular component | *GNA13*, *ATP8A1*, *LDB2*, *FGF10* | 4.57E-02 |
| GOTERM_BP_FAT | GO:0001942 | Hair follicle development | *LDB2*, *FGF10* | 4.65E-02 |
| GOTERM_BP_FAT | GO:0022404 | Molting cycle process | *LDB2*, *FGF10* | 4.65E-02 |
| GOTERM_BP_FAT | GO:0022405 | Hair cycle process | *LDB2*, *FGF10* | 4.65E-02 |
| GOTERM_BP_FAT | GO:0098773 | Skin epidermis development | *LDB2*, *FGF10* | 4.81E-02 |
| GOTERM_BP_FAT | GO:0042633 | Hair cycle | *LDB2*, *FGF10* | 5.04E-02 |
| GOTERM_BP_FAT | GO:0042303 | Molting cycle | *LDB2*, *FGF10* | 5.04E-02 |
| GOTERM_BP_FAT | GO:0045944 | Positive regulation of transcription from RNA polymerase II promoter | *LDB2*, *PPARGC1A*, *FGF10* | 5.66E-02 |
| GOTERM_BP_FAT | GO:0019827 | Stem cell population maintenance | *LDB2*, *FGF10* | 6.83E-02 |
| GOTERM_BP_FAT | GO:0098727 | Maintenance of cell number | *LDB2*, *FGF10* | 6.98E-02 |
| GOTERM_BP_FAT | GO:0048754 | Branching morphogenesis of an epithelial tube | *GNA13*, *FGF10* | 9.11E-02 |
| INTERPRO | IPR024079 | Metallopeptidase, catalytic domain | *AMZ2*, *LOC100358067* | 7.48E-02 |


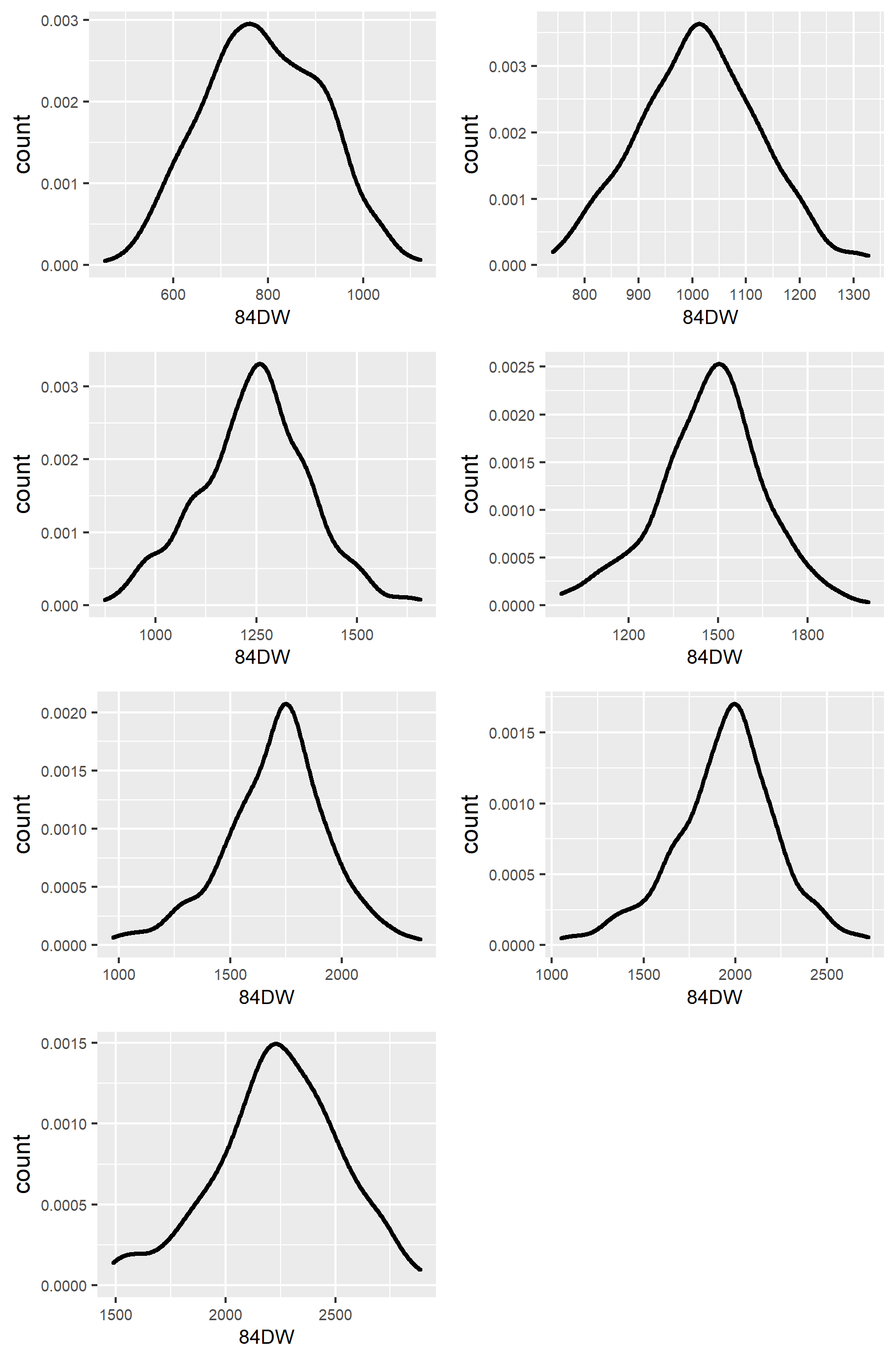


**Supplementary Figure S1. Histogram of the frequency distribution of body weight in seven growth stages.** DW = body weight on the test day.


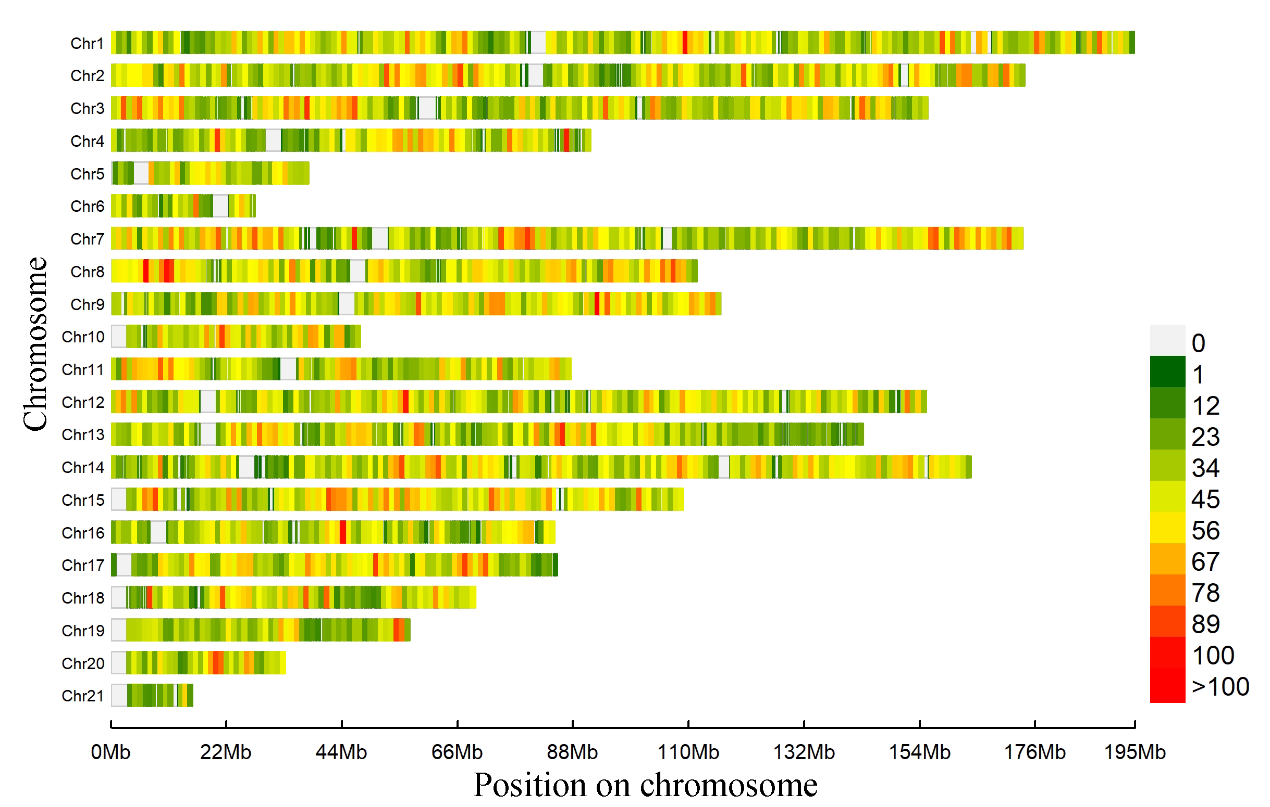


**Supplementary Figure S2. Density distribution of SNPs on 21 autosomes after performing the quality controls.**


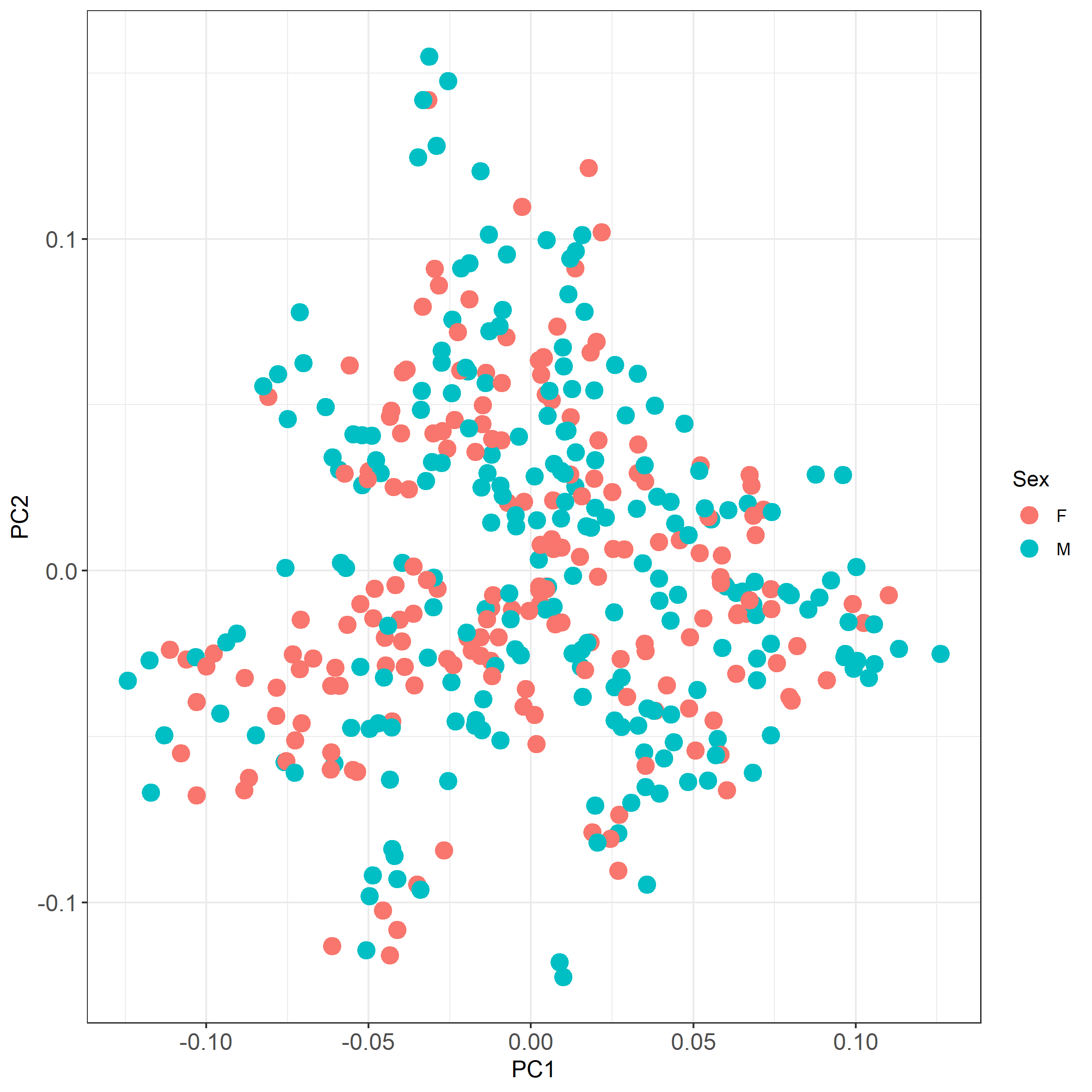


**Supplementary Figure S3. Principal component analysis of population structure.**
